# Increasing accuracy of genomic selection in presence of high density marker panels through the prioritization of relevant polymorphisms

**DOI:** 10.1186/s12863-019-0720-5

**Published:** 2019-02-22

**Authors:** Ling-Yun Chang, Sajjad Toghiani, Samuel E. Aggrey, Romdhane Rekaya

**Affiliations:** 1 Department of Animal and Dairy Science, University of Georgia, Athens, GA 30602 USA; 2 Department of Poultry Science, University of Georgia, Athens, GA 30602 USA; 3 Institute of Bioinformatics, University of Georgia, Athens, GA 30602 USA; 4 ABS Global, Inc., DeForest, WI 53532 USA; 5 USDA Agricultural Research Service, Fort Keogh Livestock and Range Research Laboratory, Miles City, MT 59301 USA

**Keywords:** Genomic selection, High density panel, SNP prioritization

## Abstract

**Background:**

It becomes clear that the increase in the density of marker panels and even the use of sequence data didn’t result in any meaningful increase in the accuracy of genomic selection (GS) using either regression (RM) or variance component (VC) approaches. This is in part due to the limitations of current methods. Association model are well over-parameterized and suffer from severe co-linearity and lack of statistical power. Even when the variant effects are not directly estimated using VC based approaches, the genomic relationships didn’t improve after the marker density exceeded a certain threshold. SNP prioritization-based fixation index (F_ST_) scores were used to track the majority of significant QTL and to reduce the dimensionality of the association model.

**Results:**

Two populations with average LD between adjacent markers of 0.3 (P1) and 0.7 (P2) were simulated. In both populations, the genomic data consisted of 400 K SNP markers distributed on 10 chromosomes. The density of simulated genomic data mimics roughly 1.2 million SNP markers in the bovine genome. The genomic relationship matrix (**G)** was calculated for each set of selected SNPs based on their F_ST_ score and similar numbers of SNPs were selected randomly for comparison. Using all 400 K SNPs, 46% of the off-diagonal elements (OD) were between − 0.01 and 0.01. The same portion was 31, 23 and 16% when 80 K, 40 K and 20 K SNPs were selected based on F_ST_ scores. For randomly selected 20 K SNP subsets, around 33% of the OD fell within the same range. Genomic similarity computed using SNPs selected based on F_ST_ scores was always higher than using the same number of SNPs selected randomly. Maximum accuracies of 0.741 and 0.828 were achieved when 20 and 10 K SNPs were selected based on F_ST_ scores in P_1_ and P_2_, respectively.

**Conclusions:**

Genomic similarity could be maximized by the decrease in the number of selected SNPs, but it also leads to a decrease in the percentage of genetic variation explained by the selected markers. Finding the balance between these two parameters could optimize the accuracy of GS in the presence of high density marker panels.

**Electronic supplementary material:**

The online version of this article (10.1186/s12863-019-0720-5) contains supplementary material, which is available to authorized users.

## Background

A large number of polymorphic variation (e.g., SNPs, rare variants) is being identified across the genome of livestock species. A continuous decrease in the costs of high-throughput genotyping and sequencing techniques has allowed for the generation of a massive amount of genomic information on a large number of individuals. This wealth of genomic information was useful in understanding the association between complex phenotypes and genetic variation with applications in human, plants and livestock species [2, 4, 15, 19, 29].

In livestock and plants, genomic information was mainly used for breeding purposes. In fact, genomic enhanced breeding values (GEBV), which are computed as a linear function of the SNP effects and their associated genotypes, were accurately estimated through the so called genomic selection (GS). The latter is superior to its pedigree-based counterpart due to a better modeling of the Mendelian sampling and a substantial reduction in generation interval. In fact, genomic selection (GS) is becoming the standard tool for genetic evaluation in several livestock and poultry species. However, the continuous increase in the density of marker panels and number of genotyped individuals and the presence of low (rare) frequency variants are posing major challenges to GS. Regression (RM) based approaches model directly the association between the phenotype and variant genotypes. The large number of unknown parameters in the association model and the high LD will undoubtably lead to noticeable shrinkage. Splitting the effect of a QTL between a large number of linked markers will negatively affect the statistical power [20, 39]. Thus, it has become a necessity to reduce the dimensionality through prioritization (selection) of variants. Several approaches including simple regression, Bayesian, and wrapper methods [11, 22, 24, 33]) were frequently used for SNP filtering. Unfortunately, their efficiency is limited mainly due to the small marker effects and high false positives. To offset the limitations of statistical methods, the use of external information was proposed to enhance the SNP prioritization process. MacLeod et al. [23] proposed the BayesRC method where existing biological information was used as external prior information. Although attractive, it did not result in an increase in accuracy compared to BayesR [17]. Chang et al. [6] proposed using population genetic parameters that can be derived from the existing marker data to enhance the prioritization process. Their F_ST_ based prioritization resulted in sight superiority compared to BayesB.

An increase in the number of variants does not directly affect the dimensionality of the association model using variance component (VC) based approaches, such as GBLUP or ssBLUP [1, 16]. However, in their current form they are unlikely to benefit from the use of information provided by high density marker panels and next generation sequencing (NGS). The superiority of GS compared to pedigree-based selection using VC approaches is due to the use of the observed (**G**) rather than the expected (**A**) additive relationship matrix which allows for the correction of erroneous and unreported pedigree information, and a better modeling of Mendelian sampling [7, 8, 18].

Several studies have clearly shown that an increase in SNP density, after a certain threshold, does not seem to affect the quality of the estimated observed relationship matrix **G**. In fact, accuracy obtained using the 777 K SNP panel was not any different from using the 54 K SNP panel [34, 35].

These challenges are further exuberated by the added computational costs. For RM approaches, the computational cost increases almost linearly with the increase in the number of genotyped animals. However, that is not the case with the increase in the number of variants which will make the approach almost impossible computationally when using sequence data. Such costs will not be reduced even when methods for variant prioritization (BayesB, BayesR) due to the cost of identifying the “relevant” variants. For VC-based approaches, the number of variants will have very little computational costs. However, the latter increases cubically with the number of genotyped animals, complicating the direct inversion of **G.** The algorithm for proven and young animals (APY) method developed by Fragomeni et al. [13, 14] to approximate the inverse of **G** is intrinsically data-driven and could result in computational problems. As a data-driven approximation, its performance is not guaranteed with a continuous increase in the number of genotyped animals, which may span several generations and have more complex pedigree structures (inbreeding). As currently implemented, the ss-GBLUP method does not benefit from high density genomic data.

Although prioritization methods based on statistical (e.g., BayesB), external prior information (e.g., BayesRC), and population genetics criteria (e.g., F_ST_) have been frequently applied in RM, little has been done to evaluate the impact of marker prioritization on the estimation of the genomic relationship matrix (**G**) and the potential impact in GS using VC approaches [12]. The latter will benefit from SNP marker prioritization for two reasons: 1) only relevant markers will be used to compute **G,** removing the contribution of non-influential SNPs that could increase or decrease the realized genetic similarity between two individuals, especially for low MAF markers; 2) some prioritization methods (e.g., based on F_ST_) could provide a simple and systematic approach for weighting the contribution of different markers to the estimation of **G**. For example, this could be accomplished by using the individual marker F_ST_ score as a weight factor. In this study, a marker prioritization method presented by Chang et al. [6] will be assessed for its impact on the estimation of the genetic similarity between individuals and on the accuracy of GS. For that purpose, SNP markers in high density panels will be prioritized using F_ST_ score as suggested by Chang et al. [6].

## Methods

### Simulation: population structure

Data was simulated to mimic high-density marker panels using the QMSim simulation software [31]. First, a historical population (HP) was generated through random mating to initialize LD and to establish mutation-drift equilibrium. The HP was used as a base to create two populations (P_1_ and P_2_) with average LD between adjacent markers of 0.3 and 0.7, respectively. Gametes were randomly sampled from both male and female gamete pools. To produce a realistic level of LD in population P_1_, 300 historical generations were generated based on random mating of an initial 8000 animals, increasing to 15,000 animals at generation 305, decreasing to 12,000 animals at generation 1000, and then increasing to 17,000 animals at the last generation. For population P_2_, the initial 8000 animals were also simulated for 300 generations but followed by an additional 5 generations with 15,000, 5 generations with 12,000, and 5 generations with 17,000 animals. In a second step, 1000 males and 15,000 females were randomly selected from the historical population and used to create the founder population (G_0_) for P1 and P2. A trait with heritability equal to 0.30 was simulated assuming that all genetic variation was due to the simulated QTL. An additional 7 selection generations (G_1_-G_7_) of 15,000 animals each were simulated. Parents were chosen based on their estimated breeding values (EBVs). The replacement rate for males and females was 50 and 20%, respectively. Throughout, one progeny per mating was assumed and the sex ratio of progeny was set to 50%. The average effective population size ranged between 323 and 350 for P_1_ and P_2_, respectively. The sixth generation (G_6_) was considered as the training population and the last generation (G_7_) was used to evaluate (validation population) our proposed method.

In both populations, only animals in the training and validation populations were genotyped. Genotypes were simulated for 400 K biallelic SNP markers uniformly-distributed along 10 chromosomes of 100 cM in length each to roughly mimic 1.2 million SNP markers in the bovine genome. Two hundred biallelic QTL were sampled from a Gamma distribution with shape parameter equal to 0. 4. No overlap between SNP markers and QTL was allowed. Additionally, QTL were assumed not to be genotyped. In general, the genotype structures for P_1_ and P_2_ were similar with the exception that P_2_ had higher LD between adjacent markers. The residual variance was scaled accordingly in each scenario of selected SNPs such that the heritability and phenotypic variance were constant at the values of 0.3 and 1, respectively. Trait phenotypes were generated as the sum of an overall mean, the random additive effects of QTL and their associated genotypes, and the residual terms. The later were sampled from a normal distribution with zero mean and variance-covariance matrices *Iσ*_*e*_^*2*^, where *σ*_*e*_^*2*^ is the residual variance.

### Real data

A real dataset consisting of weaning weight (WW) records of 3012 animals from a composite beef cattle breed born between 2002 and 2011 at the USDA-ARS, Fort Keogh Livestock and Range Research Laboratory, Miles City, MT [27, 28] was used. The mean and standard deviation of WW records were 209.58 and 30.73 kg, respectively. The systematic effects associated with this data consisted of sex (2 classes), feeding treatment (2 classes), year of birth (10 classes) and three covariates: age of dam, age at the weaning weight, and birth weight. The pedigree file included 5374 animals.

These animals were genotyped with a mixture of different density SNP commercial arrays. Only SNPs with call rate greater than 0.90, minor allele frequency (MAF) greater than 0.05, and heterozygous deviation smaller than 15% from Hardy-Weinberg Equilibrium (HWE) were kept. Animals with call rate less than 0.90 were discarded. Animals genotyped with low-density panels were imputed to the 50 K SNP array using FImpute software [30]. The same QC process was reapplied after imputation. The final dataset consisted of 2193 animals genotyped for 41,694 SNP markers. A five-fold cross validation (80% training set and 20% validation set) was used in the analysis of the real data.

### Method of prioritizing SNPs: F_ST_ approach

Wright’s fixation indexes, F_ST_ in particular, have been used to measure the level of variation among subpopulations with respect to the variation in the total population. F_ST_ measures genetic differentiation through the change in allele frequencies among groups. The greater the divergence between subpopulation, the larger are the F_ST_ scores. In this study, F_ST_ scores were calculated following the estimators presented by Nei [26] and Chang et al. [6]. Briefly, animals in generation 6 (G_6_) were grouped into three sub-populations (below the 5% quantile [S1], between 5 and 95% quantiles [S0], and above the 95% quantile [S2]) based on the distribution of their phenotypes. Genotypes of individuals in sub-populations S1 and S2 (1500) were used to calculate the F_ST_ scores. For each locus, the global F_ST_ estimator was defined as:

$$ {F}_{ST}=\frac{H_T-{H}_S}{H_T} $$

$$ \mathrm{with}\kern0.5em {H}_T\kern0.5em =\kern0.5em {2}^{\ast }{p}^{\ast}\kern0.5em q,\kern0.5em {H}_{S\kern0.5em }=\kern0.5em \frac{{H_{S1\kern0.5em }}^{\ast}\kern0.5em {n}_{S1\kern0.5em }+\kern0.5em {H_{S2}}^{\ast}\kern0.5em {n}_{S2}}{n_{S1}\kern0.5em +\kern0.5em {n}_{S2}},\kern0.5em \mathrm{and}\kern0.5em {H}_{Si\kern0.5em }\kern0.5em =\kern0.5em {2}^{\ast}\kern0.5em {p_{Si}}^{\ast}\kern0.5em {q}_{Si} $$

where, *p*_*Si*_ and *q*_*Si*_ are the allele frequencies in subpopulation *i*, *n*_*s*1_ and *n*_*s*2_ are the number of individuals of the subpopulations, *H*_*S*_ is the average of sub-population heterozygosities and *H*_*T*_ is the heterozygosity based on the total population.

### Genetic similarity

Historically, genetic similarity between individuals is measured by their average expected additive relationships derived from pedigrees. With the availability of genetic markers, SNP panels with reasonable density provide an alternative tool to estimate genetic similarity based on realized relationships or other measurements. Currently, genomic relationships are calculated based on identity by state (IBS) between alleles of SNP markers [37]. It basically measures the similarity of marker genotypes between two individuals at a large number of loci independently of their mode of inheritance. Although estimated realized relationships using IBS are in general better than pedigree-based estimates, they still suffer from several problems, including the non-zero estimates of realized relationship between two individuals that are not related by ancestry [3, 9, 21] and the inevitable noise associated with these estimates. More importantly though is that as the SNP marker density increases, after a certain threshold, it seems not to affect the quality of the estimated observed relationships. The accuracy obtained using the 777 K SNP panel is not different from using the 54 K SNP panel [34]. This is because the 777 K panel did not improve the quality of realized genomic relationships in any significant way. Thus, in the presence of high density marker data, using all SNPs to estimate genetic similarity will not improve the genomic relationships. To the contrary, it could lead to less accurate estimates of genetic similarity. This clearly indicates that true genetic relationships could be accurately estimated by a reasonably small number of well distributed SNP markers. From genomic selection perspectives, the lack of improvement in accuracy using high density panels is not due to the lack of useful information in the additional marker genotypes, rather, it is due to the limitations of current methods. This functional similarity will likely be higher than the standard additive relationships from all SNPs if it is calculated based on a selected subset of SNPs prioritized based on their ability to increase genetic or phenotypic similarity between individuals. As the marker density increases, especially in the presence of SNPs with low minor allele frequency, prioritization of SNP markers to be included in the calculation of the genomic similarity becomes more relevant. This is the case because as the number of SNPs increases, the genomic relationships move closer to the expected relationships. Furthermore, variants with rare allele frequencies will have a very limited influence on the calculation of **G,** as most of the animals will have the same genotype (homozygous major). Using prioritized SNPs, it is likely to result in an increase in genomic similarity between individuals of similar genetic values or phenotypes. This is the case as individuals with dissimilar genetic values or phenotypes are likely to have much lower genomic sharing. Genomic similarity between two individuals (***i*** and ***j***) was calculated as:

1 $$ sim\ \left(i,j\right)=\frac{1}{2n}{\sum}_{k=1}^n{S}_k\left(i,j\right) $$

where *S*_*k*_(*i*, *j*) is the number of IBS shared alleles between individuals *i* and *j* at locus *k*. Genetic similarity was computed based on all SNPs in the panel and subsets of 2.5, 5, 10, 20, 40, 80 and 160 K markers selected either based on F_ST_ scores or at random.

### Statistical model and data analysis

For both simulated populations (P_1_ and P_2_), 10,000 and 5000 animals were randomly selected from G6 and G7, respectively. For each population, several data sets with different number of SNPs (from 10 to 400 K) selected either using F_ST_ scores or at random were generated. Data was analyzed using the following mixed linear model:

$$ \boldsymbol{y}=\boldsymbol{Xb}+\boldsymbol{Zu}+\boldsymbol{e} $$

Where ***y*** is the vector of phenotypes, ***b*** is the vector of fixed effects, ***u*** is the vector of genomic breeding values, and ***e*** is the vector of random residuals. ***X*** and ***Z*** are known incidence matrices with the appropriate dimensions. Additionally, it was assumed that $\boldsymbol{u}\sim N\left(\mathbf{0},\mathbf{G}{\sigma}_u^2\right)$ where **G** is the genomic relationship matrix and ${\sigma}_u^2$ is the genetic variance.

AIREMLF90 program, a modification of restricted maximum likelihood (REML) approach with the Average-Information algorithm [25], was used to estimate variance components and genomic breeding values under the different scenarios. Accuracy of genomic evaluation was defined as the correlation between true breeding value and the genomic estimated breeding value in validation population. In this study, each simulation scenario was replicated 5 times.

## Results

**Fig. 1:**
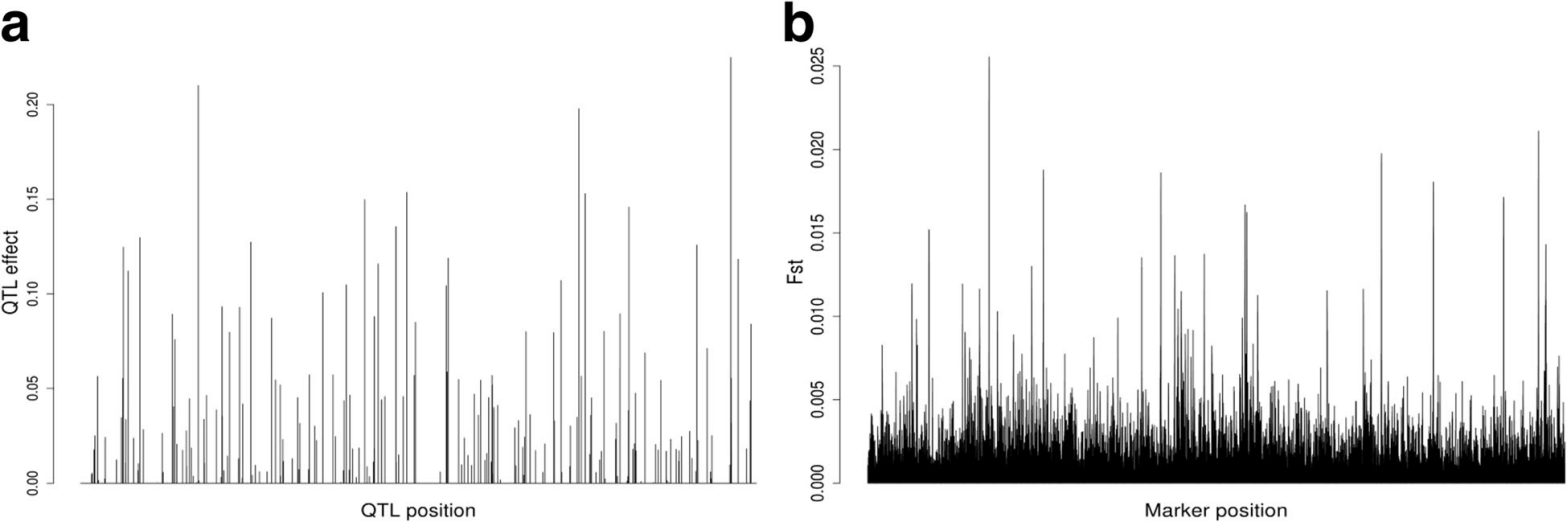
Effects and distribution of the 200 simulated quantitative trait loci (QTL) along the ten chromosomes (**a**) and their associated F_ST_ scores distribution (**b**) when the LD between adjacent markers was equal to 0.7

**Fig. 2:**
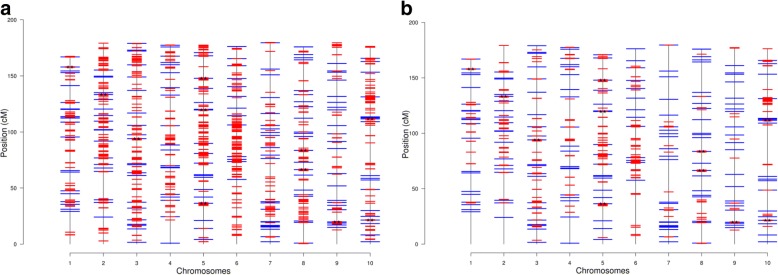
Distribution of the 200 simulated QTL (in blue) and 10 K (**a**) and 5 K (**b**) preselected SNPs based on F_ST_ scores (in red) across the 10 chromosomes when LD between adjacent markers was equal to 0.7 (* indicates the top 10% QTL)

**Fig. 3:**
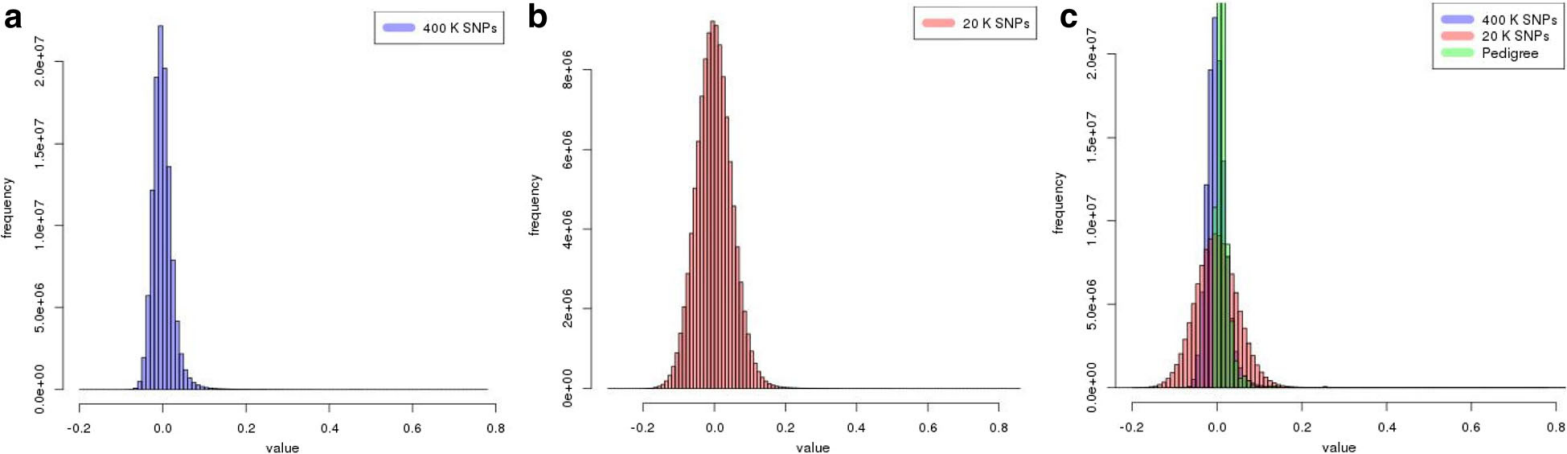
Distribution of off-diagonal elements of the additive relationship matrix using **a**) all 400 K SNP markers (in blue), **b**) 20 K SNPs prioritized based on their F_ST_ scores (in red), and **c**) pedigree information (in green)

The distribution and effects of simulated 200 QTL are presented in Fig. 1a, and the estimated F_ST_ scores of the 400 K SNPS are shown in Fig. 1b for the scenario when the LD between adjacent markers equals 0.7 (Additional file 1: Figure S1 represents the results for population P_1_). Subsequently, the distribution of simulated QTL across the 10 chromosomes based on their F_ST_ score for population P_2_ (LD = 0.7) for the top 10 K and 5 K selected SNPs, are represented in Fig. 2a and b, respectively. The estimates of functional genomic similarity based on the number of selected SNPs are presented in Table 1 and the relationship between the matrices **G** and **A** when selected subsets of SNPs are used is shown in Table 2 and Fig. 3. Estimates of the variance components and their associated standard deviations under different scenarios of preselection SNP markers used to compute **G** are presented in Table 3. Using all SNPs in the 400 K panel resulted in genomic accuracy of 0.716 and 0.760 for P_1_ and P_2_, respectively (Table 4). When SNPs were prioritized based on their F_ST_ scores, accuracy ranged between 0.723 to 0.741, and 0.784 to 0.828 for P_1_ and P_2_, respectively (Table 4). The results of the analysis of real data under different numbers of selected SNPs are presented in Table 5.

**Table 1:** Functional genomic similarity under different subsets of F_ST_ based and randomly selected SNPs for the scenario when LD^a^ between adjacent markers was equal to 0.7. Standard errors of Functional genomic similarity are listed between parentheses

	Genomic similarity	
SNPs	F_ST_ based	Random
2.5 K	0.7013 (0.0020)	0.6695 (0.0003)
5 K	0.6862 (0.0020)	0.6687 (0.0003)
10 K	0.6752 (0.0010)	0.6682 (0.0002)
20 K	0.6718 (0.0006)	0.6678 (0.0001)
40K	0.6712 (0.0005)	0.6675 (0.0001)
80 K	0.6708 (0.0004)	0.6673 (0.0001)
160 K	0.6705 (0.0003)	0.6672 (0.0001)
400 K	0.6671 (0.0003)	0.6671 (0.0001)

^a^*LD* linkage disequilibrium

**Table 2:** Distribution of off-diagonal elements (OD) of the genomic relationships matrix corresponding to the training and validation individuals under different selection criteria of SNP markers (in %)

	20 K SNPs		40 K SNPs		80 K SNPs		400 K SNPs	Pedigree
	S^1^	R^2^	S	R	S	R	-	-
OD < -0.05	15.47	1.79	7.30	1.64	2.42	0.66	0.11	0
-0.05 < OD < - 0.03	11.71	8.80	11.97	8.56	9.54	6.35	3.30	0
-0.03 < OD < - 0.01	14.96	23.79	19.60	23.93	23.16	24.72	24.43	0
-0.01 < OD < 0.01	16.19	32.57	22.98	33.1	30.78	37.96	45.91	60.09
0.01 < OD < 0.03	14.85	22.75	19.98	22.85	22.46	23.39	22.72	32.55
0.03 < OD < 0.05	11.54	8.26	11.62	8.02	9.09	5.96	3.15	5.25
OD > 0.05	15.28	2.04	7.25	1.90	2.56	0.95	0.39	2.11

^1^SNPs selected based on F_ST_scores; ^2^SNPs randomly selected

**Table 3:** Variance component estimates (standard deviation) under different subsets of F_ST_based and randomly selected SNPs for populations^1^P_1_and P_2_(average over 5 replicates)

	P_1_(LD =0.3)		P_2_(LD = 0.7)	
	GV^2^	RV^3^	GV	RV
F_ST_ based				
2.5 K	0.126 (0.017)	0.728 (0.027)	0.198 (0.029)	0.736 (0.006)
5 K	0.149 (0.016)	0.706 (0.030)	0.204 (0.005)	0.711 (0.001)
10 K	0.175 (0.023)	0.684 (0.037)	0.195 (0.009)	0.697 (0.004)
20 K	0.203 (0.031)	0.663 (0.044)	0.195 (0.007)	0.686 (0.007)
40 K	0.226 (0.041)	0.649 (0.052)	0.203 (0.009)	0.677 (0.007)
80 K	0.247 (0.048)	0.641 (0.055)	0.217 (0.008)	0.671 (0.008)
160 K	0.264 (0.045)	0.642 (0.047)	0.235 (0.008)	0.670 (0.008)
Random				
2.5 K	0.104 (0.013)	0.834 (0.012)	0.155 (0.012)	0.788 (0.006)
5 K	0.139 (0.016)	0.796 (0.013)	0.185 (0.013)	0.757 (0.005)
10 K	0.173 (0.019)	0.762 (0.006)	0.215 (0.011)	0.730 (0.012)
20 K	0.203 (0.023)	0.733 (0.013)	0.234 (0.010)	0.712 (0.008)
40 K	0.227 (0.026)	0.710 (0.015)	0.242 (0.007)	0.703 (0.005)
80 K	0.238 (0.027)	0.770 (0.015)	0.246 (0.008)	0.699 (0.007)
160 K	0.242 (0.027)	0.696 (0.016)	0.250 (0.008)	0.696 (0.006)
Full panel				
400 K	0.247 (0.027)	0.692 (0.016)	0.251 (0.007)	0.695 (0.006)

^1^P_1_: 200 QTLs and linkage disequilibrium (LD) between adjacent markers equal to 0.3 and P_2_: 200 QTLs and LD between adjacent markers equal to 0.7; ^2^genetic variance, ^3^residual variance

**Table 4:** Accuracy of genomic prediction (standard deviation) under different subsets of F_ST_ based and randomly selected SNPs for populations^a^ P_1_ and P_2_ (average over 5 replicates)

	Accuracy^b^	
	P_1_ (LD = 0.3)	P_2_ (LD = 0.7)
F_ST_ based		
2.5 K	0.724 (0.021)	0.805 (0.014)
5 K	0.736 (0.022)	0.823 (0.012)
10 K	0.740 (0.023)	0.828 (0.013)
20 K	0.741 (0.027)	0.824 (0.013)
40 K	0.735 (0.027)	0.815 (0.014)
80 K	0.728 (0.028)	0.802 (0.012)
160 K	0.723 (0.031)	0.784 (0.013)
Random		
2.5 K	0.600 (0.054)	0.669 (0.019)
5 K	0.640 (0.047)	0.709 (0.015)
10 K	0.676 (0.036)	0.736 (0.019)
20 K	0.695 (0.037)	0.746 (0.014)
40 K	0.707 (0.034)	0.754 (0.010)
80 K	0.712 (0.033)	0.757 (0.013)
160 K	0.715 (0.031)	0.759 (0.011)
Full panel		
400 K	0.716 (0.032)	0.760 (0.011)

^a^P_1_: 200 QTLs and linkage disequilibrium (LD) between adjacent markers equal to 0.3 and P_2_: 200 QTLs and LD between adjacent markers equal to 0.7;^b^ correlation between true and predicted breeding values

**Table 5:** Variance component estimates, accuracy of genomic prediction, and heritability (standard deviation) under different subsets of F_ST_ based and randomly selected SNPs for weaning weight of beef cattle

	Accuracy^a^	GV^b^	RV^c^	Heritability
F_ST_ based				
2.5 K	0.36 (0.02)	91.39 (6.28)	321.41 (7.43)	0.22 (0.01)
5 K	0.36 (0.02)	119.32 (8.67)	299.13 (7.87)	0.29 (0.02)
20 K	0.33 (0.03)	144.94 (15.74)	286.10 (11.88)	0.34 (0.03)
Random				
2.5 K	0.26 (0.04)	83.75 (13.99)	346.11 (12.57)	0.19 (0.03)
5 K	0.25 (0.03)	100.34 (17.60)	332.13 (15.87)	0.23 (0.04)
20 K	0.27 (0.01)	120.67 (15.30)	313.64 (11.87)	0.28 (0.03)
Full panel				
50 K	0.27 (0.02)	128.08 (17.86)	306.69 (13.33)	0.29 (0.04)

^a^correlation between adjusted phenotypes and predicted breeding values;^b^ genetic variance,^c^ residual variance

## Discussion

### Distribution of QTL and estimated F_ST_ values

The efficiency of a marker prioritization method depends on its ability to track all the QTL controlling a trait and in a worst case scenario it should track the most influential ones. Similar to the results obtained by Toghiani et al. [36] and Chang et al. [6], there is a striking similarity between the distribution of QTL effects and estimated F_ST_ scores. In fact, there was an almost perfect overlap between the peaks in Fig. 1a (QTL with large effects) and Fig. 1b (SNPs with large F_ST_ scores). This overlap persists even for QTL with moderate to small effects indicating the ability of F_ST_ scores to track the distribution and effects of the majority of simulated QTL. Obviously, the ability to track QTL using F_ST_ scores depends primarily on the heritability of the trait, the genetic variance explained by the QTL, the population structure, and LD between markers and QTL and among markers. For population P_2_, 50, 27 and 21% of QTL explained less than 0.1, between 1 and 0.1 and greater than 1% of genetic variance each. Similar percentages were observed for population P_1_. Although this distribution of QTL effects is unlikely in human populations, it is not unexpected in highly selected plant and animal populations. From the distribution of simulated QTL across the 10 chromosomes (Fig. 2a and Fig. 2b) it is clear that the majority of QTL are tracked by more than one SNP and only QTL with very small effects (< 0.01% of genetic variance) were not effectively tracked (e.g., first QTL in the lower end of chromosome 4). Over 85 and 78% of the genetic variance was tracked by the 10 K and 5 K preselected SNPs, respectively.

### Dissection of genomic relationship matrix and genetic similarity

The functional genomic similarity based on different number of selected SNP is presented in Table 1. Under the random scenario, genomic similarity was the same across the different SNP densities and when all 400 K markers were used. This is in line with the limited improvement in the estimation of the genomic relationships with the increase of marker density [11, 34, 38]. However, when SNPs were prioritized based on their F_ST_ scores, functional similarity increased with the decrease in the number of selected markers and was higher than its counterpart in the random selection scenario. Prioritization based on F_ST_ scores resulted in a 0.5 to 1.5% increase in genetic similarity across the different marker densities (Table 1).

When constructed from a sufficiently large number of randomly selected markers, the **G** matrix is a good estimator of the true additive relationships between individuals [5, 10]. However, when a subset of markers selected based on F_ST_ scores is used to compute **G**, the resulting matrix tends to maximize the association between phenotypes and genotypes rather than to estimate additive relationships between individuals. Thus, it is expected that **G** computed based on all markers, or even a subset of randomly selected markers will be closer to the pedigree-based kinship matrix (**A**) than when markers are preselected based on F_ST_ scores. This is clearly shown in Table 2. This is the case because the contribution of a SNP marker to the estimation of **G** is intrinsically weighted by its MAF, not the magnitude of its effect. Thus, after a certain threshold on the number of SNP markers is reached, little to no improvement is expected in **G** and ultimately in the performance of the association model with additional markers. The limited change in **G** with additional markers could be an indicator of the sufficiency of available SNPs in estimating the realized relationships. However, such sufficiency is not a guarantee of the optimality of the matrix for the implementation of association analyses. In fact, as the number of randomly selected SNPs increased from 20 K to 400 K, the matrix **G** gets closer to the expected additive relationship matrix (**A**) as indicated in Table 2. The matrix **G** computed based on a selected subset of 20 K markers is markedly different from **A**, especially in the tails of the distribution of off-diagonal elements indicating higher genetic similarity between individuals (Fig. 3). More importantly, larger genomic similarities between training and validation individuals were observed when subsets of SNP markers were selected based on F_ST_ scores (Table 2). The portion of genomic relationships between training and validation individuals exceeding 0.05 in absolute value ranged between 0.50 and 3.83% when all 400 K or random subsets (80 K, 40 K and 20 K) of SNPs were used. The same portion was 4.98, 14.55 and 30.75% when 80 K, 40 K and 20 K SNPs were selected based on F_ST_ scores (Table 2) and it was statistically different from the previous one (*p* < 0.05).

### Variance components and accuracy of estimated breeding value

As expected, the percentage of the genetic variance recovered increased with the increase in the number of SNPs used to compute **G** for both populations P_1_ and P_2_ (Table 3). When the LD between adjacent markers was equal to 0.3 (population P_1_), less than half of the genetic variance was recovered when **G** was estimated based on 2.5 K SNPs selected either randomly or using F_ST_ scores. The percentage increased steadily to reach a maximum when all 400 K SNP markers were used, at which point over 83% of the genetic variance was recovered. The inability to recover all the genetic variance in this case is due to the large number of QTL with very small effects. In fact, 55% of QTL have a true effect smaller than one tenth of 1 % and an additional 20% of QTL have an effect smaller than 0.5% of the total genetic variance. These small QTL are hard to track effectively when the LD is moderate to low. Although the general trend was similar when LD was set equal to 0.7 (population P_2_), the percentage of genetic variance explained for a given number of SNPs was in general higher than in P_1_ (Table 3). This is especially the case for the random selection scenario and when the number of SNPs used to estimate **G** was small for the F_ST_ score-based selection approach. Estimates of the residual variance were almost identical to the true value (0.7) when all 400 K SNPs were used to compute **G**. For the random selection scenario, there was an over-estimation of the residual variance, except for the case when 160 K SNPs were used (Table 3). This is largely due to under estimation of the genetic variance. When SNPs were prioritized based on their F_ST_ scores, the residual variance is over-estimated when the number of markers used to calculate **G** was small (< 5 K) and under-estimated when the number of markers exceed 40 K. In between these two numbers of selected SNPs, the residual variance was precisely estimated (Table 3).

The genomic accuracy was 0.716 and 0.70 for P1 and P2, respectively when all 400 K SNP panel was used (Table 4). Genomic selection relies on the assumption that QTL are in LD with at least one of the SNPs in the panel. Thus, the higher accuracy in P_2_ is due to the increase in LD between adjacent SNP markers and ultimately between markers and QTL. Across all random subsets (2.5 to 160 K SNPs), accuracy increased with the increase of the number of selected SNPs under both 0.3 and 0.7 LD scenarios. Further, accuracy was always smaller than when all 400 K SNPs were used (Table 4). When SNPs were prioritized based on their F_ST_ scores, accuracy ranged between 0.723 to 0.741, and 0.784 to 0.828 for P_1_ and P_2_, respectively (Table 4). However, accuracy did not increase continuously with the increase in the number of selected SNPs. Accuracy reached a maximum of 0.741 and 0.828 at around 20 and 10 K selected SNPs for P_1_ and P_2_, respectively. This intermediate optimum behavior of the accuracy seems to be the result of a balancing act between the percentage of the genetic variance explained by the selected SNPs and the resulting genetic similarity between individuals based on those markers. An increase in the number of prioritized SNPs will increase the percentage of the captured genetic variance (Table 3) and will ultimately result in higher accuracy. However, such an increase in the number of selected SNPs will reduce the genetic similarity between individuals in the training and validation sets (Table 1), which will lead to a reduction of accuracy. At some point, the benefits resulting from the increase in the percentage of captured genetic variance will not offset the cost (loss of accuracy) due to the reduction in genetic similarity. This behavior does not occur in the random selection scenario due to the minimal change in the genetic similarity with the increase in the number of SNPs (Table 1). Thus, accuracy is largely under the control of the percentage of captured genetic variance.

### Analysis of real data

When all 50 K SNPs were used to compute G, accuracy, defined as the correlation between the estimated genomic breeding values and the corrected phenotypes (adjusted for the estimated fixed effects) was equal to 0.27. When 2.5, 5, or 20 K SNPs were randomly selected, accuracy ranged between 0.25 and 0.27 (Table 5). However, when the same numbers of markers were prioritized using F_ST_ scores, accuracy was sustainably higher and ranged between 0.33 and 0.36. Similarly, estimates of heritability tended to be higher when SNPs were prioritized using F_ST_ scores. Except for the cases when 2.5 K SNPs were prioritized, estimates of heritability were within the range of the values reported in the literature for the trait [32]. Based on the results in Table 5, the proposed prioritization method seems to have maintained its superiority using real data.

## Conclusions

High-density SNP panels and whole genome sequence data were expected to increase the accuracy of genomic selection in livestock. However, because of the limitations of current methods used for implementation, an increase in genomic data did not result in any significant improvement of accuracy. The dramatic increase in the dimensionality of the association models led to an over-parameterization problem, such as increased co-linearity and lack of statistical power. F_ST_, a measure of genetic differentiation, was used as an additional source of information to prioritize SNPs in high-density marker panels. Prioritized markers based on F_ST_ under different scenarios were able to track the majority of significant QTL and to increase the functional genetic similarity between individuals. The latter could be maximized by the decrease in the number of selected SNPs. Unfortunately, that will lead to a reduction in the percentage of genetic variation explained by the selected markers. Thus, a balance between these two parameters is needed in order to maximize the accuracy of GS in the presence of high density marker panels. This balance is likely to depend on the heritability of the trait and its genetic complexity. However, given the simplicity and flexibility of marker prioritization using F_ST_ the balance could be easily identified empirically. As clearly shown in this study, accuracy of genomic selection could be increased using high density marker data and new implementation methods. As high density and sequence data become more common, alternative methods, including the approach presented in this study, will be needed to fully harness the benefit of genomic selection. However, several issues including marker prioritization in the presence of multiple continuous and discrete traits and their relative weights need to be addressed. Furthermore, F_ST_ prioritization could be used in conjunction with other approaches (e.g. hybrid models) to further enhance the accuracy of genomic selection.

## Additional file

Additional file 1: **Figure S1.** Effects and distribution of the 200 simulated quantitative trait loci (QTL) along the ten chromosomes (a) and their associated F_ST_ scores distribution (b) when the LD between adjacent markers was equal to 0.3 (DOCX 1094 kb)

## Abbreviations

A: Pedigree-based kinship matrix
APY: Algorithm for proven and young
BLUP: Best linear unbiased prediction
EBVs: Estimated breeding values
G: Genomic relationship matrix
GEBV: Genomic enhanced breeding values
GS: Genomic selection
GV: Genetic variance
GWAS: Genome-wide association study
IBS: Identity by state
LD: Linkage disequilibrium
MAF: Minor allele frequency
NGS: Next generation sequencing
OD: Off-diagonal element
QTL: Quantitative trait locus
REML: Restricted maximum likelihood
RM: Multiple regression
SNPs: Single nucleotide polymorphisms
VC: Variance component
