# Supplementary material for: Increasing accuracy of genomic selection in presence of high density marker panels through the prioritization of relevant polymorphisms
Source: BMC Genet. 2019 Feb 22;20:21. doi: 10.1186/s12863-019-0720-5 (PMC6387489; doi:10.1186/s12863-019-0720-5)
Supplement: Supplementary file 1 — Figure S1. Effects and distribution of the 200 simulated quantitative trait loci (QTL) along the ten chromosomes (a) and their associated FST scores distribution (b) when the LD between adjacent markers was equal to 0.3 (DOCX 1094 kb) [file 12863_2019_720_MOESM1_ESM.docx]

1. (b)
